# Transcriptional Regulation of Two Conceptus Interferon Tau Genes Expressed in Japanese Black Cattle during Peri-Implantation Period

**DOI:** 10.1371/journal.pone.0080427

**Published:** 2013-11-27

**Authors:** Toshihiro Sakurai, So Nakagawa, Min-Su Kim, Hanako Bai, Rulan Bai, Junyou Li, Kwan-Sik Min, Atsushi Ideta, Yoshito Aoyagi, Kazuhiko Imakawa

**Affiliations:** 1 Laboratory of Animal Breeding, Graduate School of Agricultural and Life Sciences, The University of Tokyo, Tokyo, Japan; 2 Department of Molecular Life Science, Tokai University School of Medicine, Kanagawa, Japan; 3 Animal Resource Center, Graduate School of Agricultural and Life Sciences, The University of Tokyo, Tokyo, Japan; 4 Animal Biotechnology, Hankyong National University, Kyonggi-do, Korea; 5 Zen-Noh ET Center, Hokkaido, Japan; University of Quebec at Trois-Rivieres, Canada

## Abstract

Interferon tau (IFNT), produced by the mononuclear trophectoderm, signals the process of maternal recognition of pregnancy in ruminants. However, its expression *in vivo* and its transcriptional regulation are not yet well characterized. Objectives of this study were to determine conceptus *IFNT* gene isoforms expressed in the bovine uterus and to identify differences in promoter sequences of *IFNT* genes that differ in their expression. RNA-seq data analysis of bovine conceptuses on days 17, 20, and 22 (day 0  =  day of estrus) detected the expression of two *IFNT* transcripts, *IFNT1* and *IFNTc1*, which were indeed classified into the *IFNT* gene clade. RNA-seq and quantitative RT-PCR analyses also revealed that the expression levels of both *IFNT* mRNAs were highest on day 17, and then decreased on days 20 and 22. Bovine ear-derived fibroblast (EF) cells, a model system commonly used for bovine *IFNT* gene transcription study in this laboratory, were cotransfected with luciferase reporter constructs carrying upstream (positions −637 to +51) regions of *IFNT1* or *IFNTc1* gene and various transcription factor expression plasmids including *CDX2*, AP-1 (*Jun*) and *ETS2*. CDX2, either alone or with the other transcription factors, markedly increased luciferase activity. The upstream regions of *IFNT1* and *IFNTc1* loci were then serially deleted or point-mutated at potential CDX-, AP-1-, and ETS-binding sites. Compared to the wild-type constructs, deletion or mutation at CDX2 or ETS2 binding sites similarly reduced the luciferase activities of *IFNT1-* or *IFNTc1*-promoter constructs. However, with the AP-1 site mutated construct, *IFNT1*- and *IFNTc1-*reporters behaved differently. These results suggest that two forms of bovine conceptus *IFNT* genes are expressed *in utero* and their transcriptional regulations differ.

## Introduction

In placental mammals, blastocysts/conceptuses must implant to the maternal endometrium and eventually develop the placenta. However, genes regulating the proper development of conceptuses and their implantation processes to the uterine endometrium have not been definitively characterized. Although artificial insemination and other assisted reproductive technologies have been developed and widely used for bovine reproduction throughout the world, the cattle industry has accomplished only a limited success in improving fertility. In fact, pregnancy rates have been declining for the last 20 years in Japan (Livestock Improvement Association of Japan, http://liaj.lin.gr.jp/japanese/chosa/index.html). This trend indicates the need to investigate other factors and/or factors that have been overlooked.

Interferons (IFNs) play a role in protective mechanisms of the organism against pathogens such as viruses. In addition, the expression of various IFNs is also found in the uterus during pregnancy. IFN tau (IFNT), in particular, is a major protein involved in the process of maternal recognition of pregnancy in ruminant ungulates [1]–[3]. Although IFNT is found only in ruminants [3], it has been categorized into type I IFNs including IFN alpha (IFNA), IFN beta (IFNB) and IFN omega (IFNW). IFNT, produced by the mononuclear trophectoderm of the conceptus, is secreted into the uterine lumen during the peri-implantation period [3], [4]. IFNT acts to decrease endometrial oxytocin receptors, which attenuates episodic prostaglandin F_2_α (PGF_2_α) secretion and in turn results in the prevention of luteolysis [5], [6]. In sheep, IFNT production is initiated on day 8, and the maximal production of IFNT is attained on day 16. The implantation process proceeds thereafter, and the IFNT expression declines rapidly. By day 22, IFNT is no longer detectable when the ovine trophoblast is fully attached to the maternal endometrium [1], [2], [7], [8]. The equivalent protein found in bovine conceptuses has also been characterized [9]–[11].

Use of IFNT products to improve pregnancy rates was developed more than two decades ago. However, no effective therapy with IFNT is known to exist. This could be due, at least in part, to the lack of information on which conceptus IFNT(s) are actively expressed *in utero*, and more importantly, on which one could be effective in eliciting the process of maternal recognition of pregnancy. Genes and cDNAs corresponding to *IFNT*s have been isolated and characterized in ovine and bovine species, which exhibit a high degree of similarity within and among ruminants [12]–[17]. The 5′-upstream regions (positions –1000 to +51) of *IFNT* genes within species share a high degree of similarity (approximately 90%) [14], [17]. However, the remaining 10% of the regulatory sequences may contain specific nucleotides that are responsible for the different degrees of expression. A careful examination to determine the number of *IFNT* genes expressed in the bovine uterus was done previously [17], [18], identifying three *IFNT* genes through the use of PCR cloning. Of these, however, two of them were confirmed to be present in more recent information of the bovine genome (Btau_4.0.55.gtf.gz). Therefore, it is still unclear as to how many *IFNT* genes are expressed *in vivo*, and whether the difference in transcriptional regulation exists.

Previously, a next generation sequencer SOLiD3 was used to identify the retroviral elements functioning for placental development in days 17, 20, and 22 bovine conceptuses [19]. In this study, rather than using bovine cDNA microarray or PCR cloning, the RNA-seq data obtained from the previous study were used to identify conceptus *IFNT* transcripts expressed *in utero*, of which promoter sequences were further examined for their ability to regulate *IFNT* gene transcription.

## Materials and Methods

### Animals and sampling

All animal use was approved by the Committee for Animal Use, Care, and Experiments at Zen-noh Embryo Transfer Center and the University of Tokyo (Permit number: P08-266). Processes of estrous synchronization, superovulation, embryo collection, and embryo transfer were performed nonsurgically as previously described [20]. Superovulated Japanese black cattle were inseminated with Holstein semen. Twelve 7-day-old embryos (day 0  =  day of estrus) collected from these cattle were transferred into the uterine horn ipsilateral to the corpus luteum of 3 Holstein heifers (4 embryos each). On days 17, 20 or 22, elongated conceptuses were collected nonsurgically by uterine flushing, centrifuged at 1,000 rpm for 5 min, and snap frozen before transfer to the Laboratory of Animal Breeding at the University of Tokyo.

### RNA extraction and RT-PCR

Total RNA (80−100 µg) was extracted from an individual conceptus on days 17, 20, and 22 (n = 3 each day) using the Isogen Reagent (Nippon Gene, Tokyo, Japan) according to the manufacturer’s protocol. For PCR and real-time PCR (qPCR) analysis, isolated RNA (1 µg) from each conceptus was reverse transcribed to cDNA using ReverTra Ace qPCR RT kit (TOYOBO, Osaka, Japan) in a 10 µl reaction volume, and the resulting cDNA (RT template) was stored at 4°C until use. The cDNA reaction mixture was diluted 1:10 using DNase- and RNase-free molecular biology grade water, of which 3 µl was taken for each amplification reaction. RT template was subjected to PCR or qPCR amplification [21]. In both cases, identical primers were used for *IFNT* transcript amplification; forward, 5′-CAGAAAAGACTTTGGTCTTCC-3′; reverse, 5′-AGAGAGGGCTCTCATCATCTC-3′. After 30 cycles of 94°C for 1 min, 57°C for 1 min, and 72°C for 1 min, amplification products were separated on 1.5% (w/v) agarose gel and were subcloned, from which 30 clones were picked and verified by DNA sequencing. qPCR reactions were performed using the SYBR Green kit (Takara Biomedicals, Tokyo, Japan) and the Applied Biosystems thermal cycle system (7900HT, Applied Biosystems, Tokyo, Japan) as described previously [21], [22]. Amplification efficiencies of each target and a reference gene, *ACTB*, were examined through their calibration curves and found to be comparable [23], [24]. The qPCR amplification consisted of 40 cycles at 95°C for 10 sec, annealing at 60°C for 20 sec, and extension at 72°C for 40 sec. The threshold cycle (Ct) value for each target was determined by Sequence Detection System software v1.2 (Applied Biosystems). Expression levels of each mRNA were normalized by calculating the Ct values based on subtracting the Ct value of target mRNA from the Ct value of the internal control, *ACTB* mRNA. Each amplification was completed with a melting curve analysis to confirm the specificity of amplification and absence of primer dimers [21].

### RNA-seq data analysis on conceptus *IFNT* transcripts

Previously, RNA-seq analysis was carried out with RNAs extracted from days 17, 20, and 22 bovine conceptuses [19], and the Applied Biosystems Whole Transcriptome Analysis Pipeline, an off-instrument SOLiD3 data analysis software package, was used to characterize the short reads. Entire read counts mapped onto the bovine genome (Btau_4.0), allowing multiple hits within 10 times, were 172,435,337, 142,294,526, and 139,083,864 for days 17, 20, and 22, respectively [19], and primary sequencing data were deposited to the DDBJ Sequence Read Archive (accession number DRA000549) [25]. Matching locations were subsequently used to generate counts for identified *IFNT*s and Ensemble-provided gene annotations (Bos_taurus. Btau_4.0.55.gtf.gz).

### Phylogenetic analysis on *IFNT* genes

Amino acid sequences of *IFNT1* (P15696.2) and *IFNTc1* (Q9GLL5) were queried with non-redundant protein database in GenBank through the use of BLASTP program, resulting in the identification of 250 hits each. These hits were merged and 268 non-redundant amino acid sequences found. Among 268 *IFNT* related genes, we utilized 218 genes that meet the following criteria: 1) start with methionine, 2) are not truncated, and 3) do not contain ambiguous sequence (Supplemental Table 1). Using L-INS-i program in an MAFFT suite [26], these sequences were aligned, from which phylogenetic trees were constructed using RAxML, a program for Maximum Likelihood-based phylogenetic inference, with 1000 times rapid bootstrapping test [27]. A JTT matrix [28] with gamma distribution for rate heterogeneity among sites for entire region (α  =  2.22) as wells as invariant proportion of replacement (*p*  =  0.02) was selected for the amino acid replacement model by ProtTest3 [29] with Akaike’s Information Criterion (AIC) scores [19].

**Table 1:** Primers for generating various *IFNT1/IFNTc1*-reporter constructs with deletions.

Name	Primer (5′→3′): Forward and Reverse
*IFNT1-637*	F: ggtacc tccctgagggccctgga
*IFNT1-389*	F: ggtacc tatgtgtaagataaggagg
*IFNT1-262*	F: ggtacc atctataagtctttgcata
*IFNT1-222*	F: ggtacc tttagtttctcatttaattgatata
*IFNT1-157*	F: ggtacc atttctactgtaaaaattaa (*Kpn*I)
	R: agatct gctgctgctgggctggct (*Bgl*II)
*IFNTc1-637*	F: ggtacc tccctgagcgccctgga
*IFNTc1-389*	F: ggtacc tatgtgtaagataaggagg
*IFNTc1-262*	F: ggtacc atctataagtctttgcata
*IFNTc1-222*	F: ggtacc tttagtttctcatttaattgatata
*IFNTc1-157*	F: ggtacc acttctactgtaaaatta (*Kpn*I)
	R: agatct gctgctgctgggctggct (*Bgl*II)

### DNA isolation and construction of *IFNT1*- and *IFNTc1*-reporter plasmids

Genomic DNA was isolated from pooled bovine conceptus tissues using the Genomic DNA Purification Kit (Promega, Madison, WI), according to the protocol provided by the manufacturer. The quality and integrity of genomic DNA was determined by agarose gel (1%) electrophoresis and visualization under UV light after ethidium bromide staining.

Isolated bovine genomic DNA was then used as the template for amplifying the *IFNT* genes. In short, the upstream regions (positions −637 to +51) of *bIFNT1* and *bIFNTc1* (GenBank accession numbers: M60903 and AF238613, respectively) were PCR amplified and then inserted into the *Kpn*I/*Nhe*I sites of pGL3 basic vector (Promega). The wild type (positions −637 to +51) and various deletion mutants (positions −389, −262, −222, and −157 to +51) were constructed using primers shown in Table 1. Several potential CDX-like sequences are located between positions −370 to −363, −301 to −292, −292 to −283, −286 to −280, −280 to −272, and −260 to −252 of the CDX binding site-rich region. Four AP-1-like sequences are located between positions −602 to −592, −444 to −435, −408 to −394, and −71 to −64, identified by others [30]. Four ETS-like sequences are located between positions −596 to −587, −573 to −565, −352 to −339, and −79 to −70 upstream of the *IFNT1* and *IFNTc1* genes. Based on the preliminary experimentations, we constructed six CDX mutants, one AP−1 mutant, and one ETS mutant by introducing a point mutation into the *IFNT*-reporters using specific primers (Table 2). Specifically, the AP-1-binding sites located between positions −602 to −592 upstream of the *IFNT1* and *IFNTc1* genes were mutated (GACTGTGTCAT to GACTGTtgCAT and GTCTCTGTCAT to GTCTCTtgCAT, respectively) by inverse PCR using primers containing desired nucleotide substitutions [31]. Similarly, the ETS-binding sites between positions −79 to −70 upstream of the *IFNT1* [11] and *IFNTc1* genes were also mutated (ACAGGAAGTG to ACAGagAGTG and CCAGGAAGTG to CCAGagAGTG, respectively). The pRL-*TK* vector (Promega), in which the *Renilla* luciferase gene is driven by the herpes simplex virus-thymidine kinase promoter, was used to normalize transfection efficiency [32]. Amounts of reporter constructs relative to those of the internal control pRL-*TK* vector were 20:1. Plasmids expressing *Cdx2*, *ETS2*, *Jun*, and *Crebbp* were described previously [32]. All reporter constructs were confirmed to have expected nucleotide sequences by dideoxy sequencing.

**Table 2:** Primers for generating *IFNT1/IFNTc1*-reporter constructs with mutations at CDX2-, AP-1-, and ETS2-binding sites.

Name	Primer (5′→3′): Forward and Reverse
*IFNT1-CDX2mt1* (−370 to −363)	F: gaagggtgcagttaagaatcaatgg
	R: cacccttccctccttatcttacac
*IFNT1-CDX2mt2* (−301 to −292)	F: tatagggtacctaaatttgtacataataac
	R: gtaccctatatataaacattccttttg
*IFNT1-CDX2mt3* (−292 to −283)	F: ccgggatttgtacataataactatgtacac
	R: caaatcccggtataatatatataaacattc
*IFNT1-CDX2mt4* (−286 to −280)	F: ctaaatgggtacataataactatgtac
	R: gtacccatttaggtataatatatataaac
*IFNT1-CDX2mt5* (−280 to −272)	F: cagggtaactatgtacacatctataag
	R: gttaccctgtacaaatttagg
*IFNT1-CDX2mt6* (−260 to −252)	F: ctaggggtctttgcatacttac
	R: gacccctagatgtgtacatag
*IFNT1-AP1mt* (−602 to −592)	F: ctgttgcatcctggtttactgatatg
	R: gatgcaacagtcatcaagagtttc
*IFNT1-ETS2mt* (−79 to −70)	F: acagagagtgagagagaaattttc
	R: cactctctgtttgtgttttcagttag
*IFNTc1-CDX2mt1* (−370 to −363)	F: aaggagggaagggtgcagttaagaatcaatggaaaa
	R: ttaactgcacccttccctccttatcttatacata
*IFNTc1-CDX2mt2* (−301 to −292)	F: ggaatgtttacatatagggtacctaaatttg
	R: caaatttaggtaccctatatgtaaacattcc
*IFNTc1-CDX2mt3* (−292 to −283)	F: tattataccgggatttgtacataataactatgta
	R: tgtacaaatcccggtataatatatgtaaacattcctt
*IFNTc1-CDX2mt4* (−286 to −280)	F: cctaaatgggtacataataactatgtacacatctataag
	R: gtgtacatagttaccatgtacccatttaggtataata
*IFNTc1-CDX2mt5* (−280 to −272)	F: cctaaatttgtacagggtaactatgtacac
	R: gtgtacatagttaccctgtacaaatttagg
*IFNTc1-CDX2mt6* (−260 to −252)	F: tacacatctaggggtctttgcatacttacataact
	R: atgcaaagacccctagatgtgtacatagtttatg
*IFNTc1-AP1mt* (−602 to −592)	F: cttgatgtctcttgcatcctggtt
	R: aaccaggatgcaagagacatcaag
*IFNTc1-ETS2mt* (−79 to −70)	F: aacacaaccagagagtgagatag
	R: ctatctcactctctggttgtgtt

### Cell culture, transient transfection, and luciferase assay

It would be ideal if bovine trophoblast cells line such as CT-1 [33] were used to study bovine *IFNT* gene transactivation. Recently, we encountered that regardless of transfection methodologies tested, CT-1 cells could not be transfected with any reporter and/or expression plasmid. Thus, bovine ear-derived fibroblast (EF) cells, obtained from biopsied ear skin of 4-month-old Japanese black bull, were used in these series of transfection experiments. This model system has been proved to be useful in studying bovine *IFNT* gene transcription [21], [22], [24]. Cells were cultured in Dulbecco's Modified Eagle Medium (DMEM, Invitrogen, Carlsbad, CA) containing 5% FBS (JRH Biosciences, Lenexa, KS) and antibiotics (Invitrogen) at 37°C in air with 5% CO_2_. For transient transfection, cells were replated onto 24-well plastic culture plates at 60−80% confluency. After 1 day, transient transfection was performed using the HilyMax Reagents (Dojin Chemicals, Kumamoto, Japan), according to the manufacturer’s protocol. In brief, 2 µg of plasmid DNA, including the *IFNT*-reporter (1.5 µg) and expression plasmids (total of 0.5 µg), along with 4 µl of HilyMax were prepared in 30 µl of DMEM with no supplements (plasmid mixture). Amounts of total plasmids for each transfection were adjusted with the inclusion of pSG5 plasmid (empty vector). After 15 min, plated cells were overlaid with the plasmid mixture and incubated at 37°C. At 48 h after transfection, cells were lysed by adding 100 µl of Passive Lysis Buffer (Promega). The luciferase assay was performed using the Dual-Luciferase Reporter Assay System as described previously [32].

### Statistical analysis

Results of luciferase assays were expressed as mean ± SEM. Differences in fold activation (luciferase activity) were examined by ANOVA, followed by multiple comparisons using Fisher’s least significant difference test.

## Results

### Expression of two *IFNT* transcripts, *IFNT1* and *IFNTc1*, *in utero* and their phylogenetic analyses

Among 35 genes that are registered as bovine *IFNT*-related genes, including *IFNT*, *IFNW*, and *IFNA* in the Ensembl database (Btau_4.0), eight genes were found to be bovine *IFNT* genes based on our phylogenetic analyses (Table S1 and Fig. S1). In the series of RNA-seq data analyses, the short reads that were mapped onto the bovine genome up to 10 multiple loci were utilized [19]. The results from days 17, 20, and 22 bovine conceptuses (DDBJ accession number DRA000549) revealed that among eight bovine *IFNT* genes, only two forms of *IFNT* transcripts, *IFNT1* (ENSBTAT00000048580, NP_001015511.3) and *IFNTc1* (ENSBTAT00000030159, DAA26985.1), were found in all of the three days examined (Table S1). The analysis was then extended for each read to be mapped onto the bovine genome up to 50 different loci, however, the same two *IFNT* genes, *IFNT1* and *IFNTc1*, were again found in all three days examined (data not shown).

**Figure 1:**
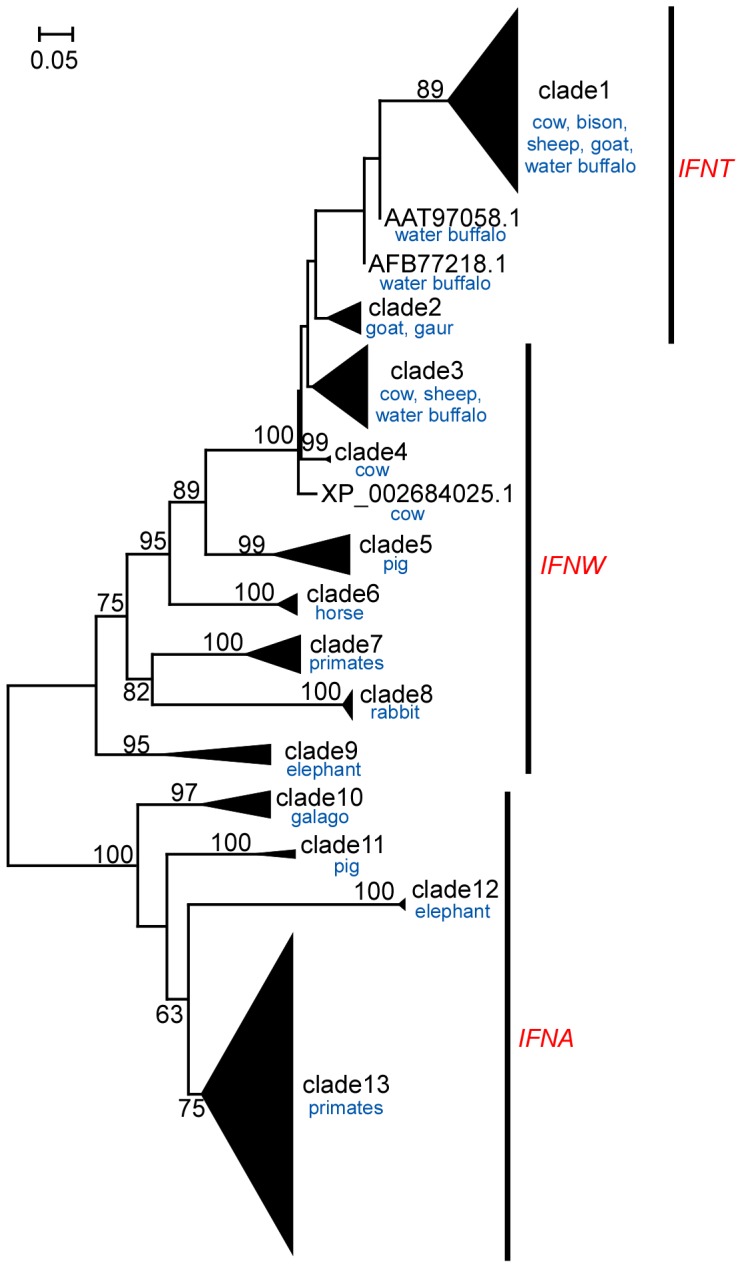
Maximum likelihood phylogenetic tree of 218 *IFNT* related genes. Amino acid sequences of 218 *IFNT* related genes were used for the phylogenetic analysis. A scale of evolutionary distance is shown in the upper left. The percentage of 1,000 fast bootstrapping tests was shown at each split. Based on values from the fast bootstrapping test as well as gene annotations, *IFNT* related sequences were divided into 13 clades, each of which is compressed in a triangle in the figure. Genes in each clade is shown in Fig. S1 and Table S2.

To investigate the evolution of these *IFNT* genes, we further conducted phylogenetic analysis using 218 *IFNT* related sequences, including *IFNT1* and *IFNTc1*, obtained by similarity searches with NCBI non-redundant database (Table S2). The 218 *IFNT* related sequences were categorized into 13 clades based on values of the rapid bootstrapping test as well as their gene annotations (Fig. 1 and Fig. S1). It should be noted that three genes, AAT97058.1 and AFB77218.1 of water buffalo and XP_002684025.1 of cow, were not categorized into any clade, and the genes in clade1 and clade2 as well as AAT97058.1 and AFB77218.1 were annotated as *IFNT* genes (Fig. 1 and Fig. S1). However, *IFNT* genes did not form a cluster and they were only clustered when combined with those of *IFNW*s. Clades of *IFNT* and *IFNW* genes were distinct from *IFNA* genes, which were supported by the high strap value (100%).

### Expression levels of *IFNT1* and *IFNTc1* mRNA in days 17, 20, and 22 conceptuses

**Figure 2:**
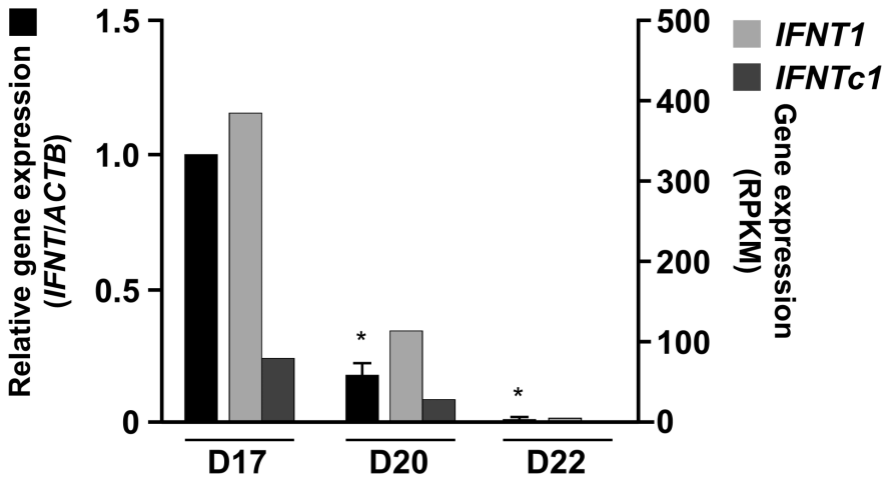
Levels of *IFNT1* and *IFNTc1* mRNAs in bovine conceptuses during early pregnancy. RNA-seq analysis was executed on RNAs extracted from days 17, 20, and 22 bovine conceptuses. Reads per kilobase of exon per million mapped reads (RPKM), number of short-tags mapped to each gene, has been corrected for the length of a gene, as previously published [19]. Real-time PCR (qPCR) analysis was then used to determine the amounts of *IFNT1* and *IFNTc1* mRNAs in days 17, 20, and 22 bovine conceptuses (n = 3 each, solid black bar on the left). RNA extracted from frozen conceptuses was subjected to qPCR analysis with primers: forward, 5′-CAGAAAAGACTTTGGTCTTCC-3′; reverse, 5′-AGAGAGGGCTCTCATCATCTC-3′. *ACTB* was used as an internal control [21], [22]. *Statistically significant differences in *IFNT* mRNA levels (*p* < 0.05) when compared to that of day 17.

*IFNT* expression decreased as the pregnancy proceeded (Table S1). Amounts of *IFNT1*, expressed as reads per kilobase of exon per million mapped reads (RPKM), were higher than those of *IFNTc1* in days 17 and 20. Because our PCR system cannot distinguish each of these *IFNT* transcripts, the primers were designed to detect both *IFNT* mRNAs. Results from both RNA-seq and quantitative PCR analyses were in agreement that *IFNT* transcripts were highest on day 17 and decreased on days 20 and 22 (Fig. 2).

### Transactivation of *IFNT1*- and *IFNTc1*-reporter constructs in EF cells

**Figure 3:**
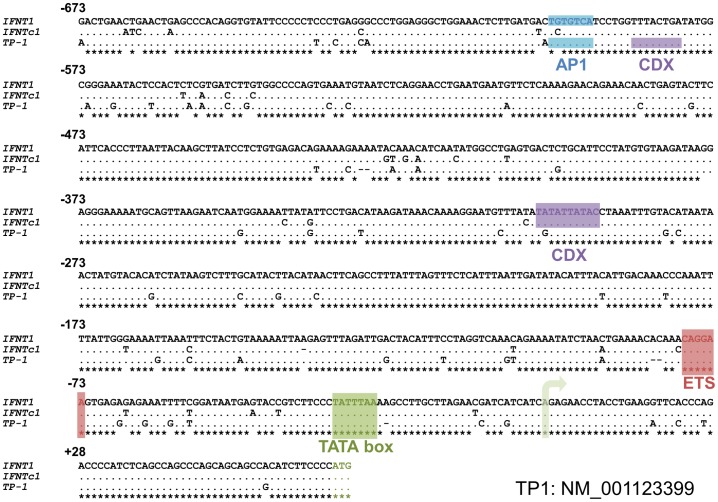
Alignments of *IFNT1* and *IFNTc1* nucleotide sequences found *in utero* and their transcription factor binding sites. Among eight *IFNT* genes registered in the bovine genome (Btau_4.0), two *IFNT* transcripts, *IFNT1* and *IFNTc1*, were found in days 17, 20, and 22 bovine conceptuses (Table S1), and their upstream regions were cloned. CDX2, ETS2, and AP-1 (JUN) binding sites on the upstream regions of *IFNT1* and *IFNTc1* genes are shown. Also shown are TATA box and transcription start site (arrow). TP-1: ovine *IFNT* gene (NM_001123399).

**Figure 4:**
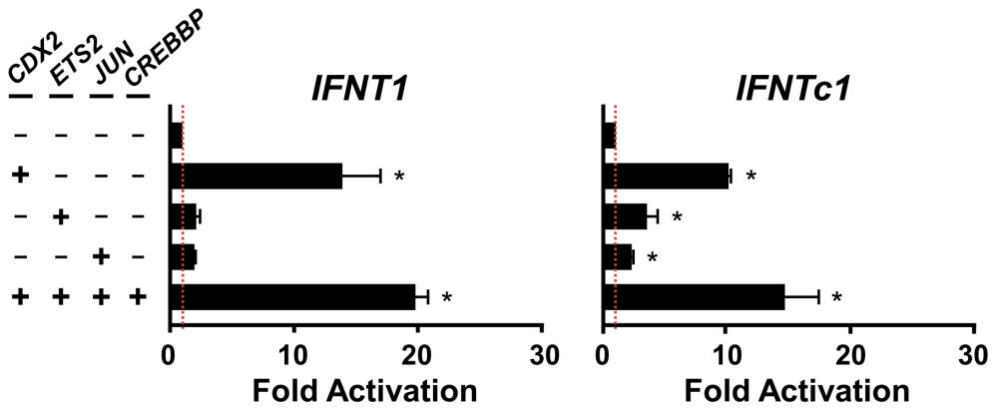
Transcriptional activity of *IFNT1* and *IFNTc1* gene promoters. The upstream regions (positions −637 to +51) of *IFNT1* (left) and *IFNTc1* (right) reporter constructs were tested in bovine ear-derived fibroblast EF cells, which have been useful for *IFNT* transcription analyses [21], [22], [24]. Each of these constructs was transfected into EF cells, either alone or with *Cdx2*, AP-1 (*Jun*), *ETS2*, and/or *Crebbp* expression plasmids. Transfection of reporter construct alone consisted of empty plasmid, resulting in the same amounts of plasmid transfection into EF cells. As a result, changes in luciferase activity were solely due to the presence or absence of expression plasmid. Values represent mean ± SEM from four independent experiments with replicate within each experiment. *Statistically significant differences in luciferase activity (*p* < 0.05) were detected when compared to that of the control (Mock: without expression plasmids).

The upstream regions for *IFNT1* and *IFNTc1* genes were cloned and their nucleotides sequenced, which were comparable to those available in the bovine genome (Fig. 3). In this study, bovine EF cells were used to characterize transcriptional regulation of *IFNT1* and *IFNTc1* genes because EF cells are a useful model for *IFNT* transcription studies [21], [22], [24]. While cotransfection with the *EST2* or AP-1 (*Jun*) expression plasmids alone had minimal effects on *IFNT*-reporter activity in EF cells, the cotransfection of the reporter construct with *Cdx2* expression plasmid, either by itself or with the other 2 or 3 transcription factors (ETS2, Jun and Crebbp), increased the luciferase activity of *IFNT1*- and *IFNTc1*-reporter constructs approximately 19 and 16-fold, respectively (Fig. 4).

### Effects of CDX2, AP-1 (JUN), or ETS2 on serially deleted *IFNT1*- and *IFNTc1*-reporter transactivation in EF cells

**Figure 5:**
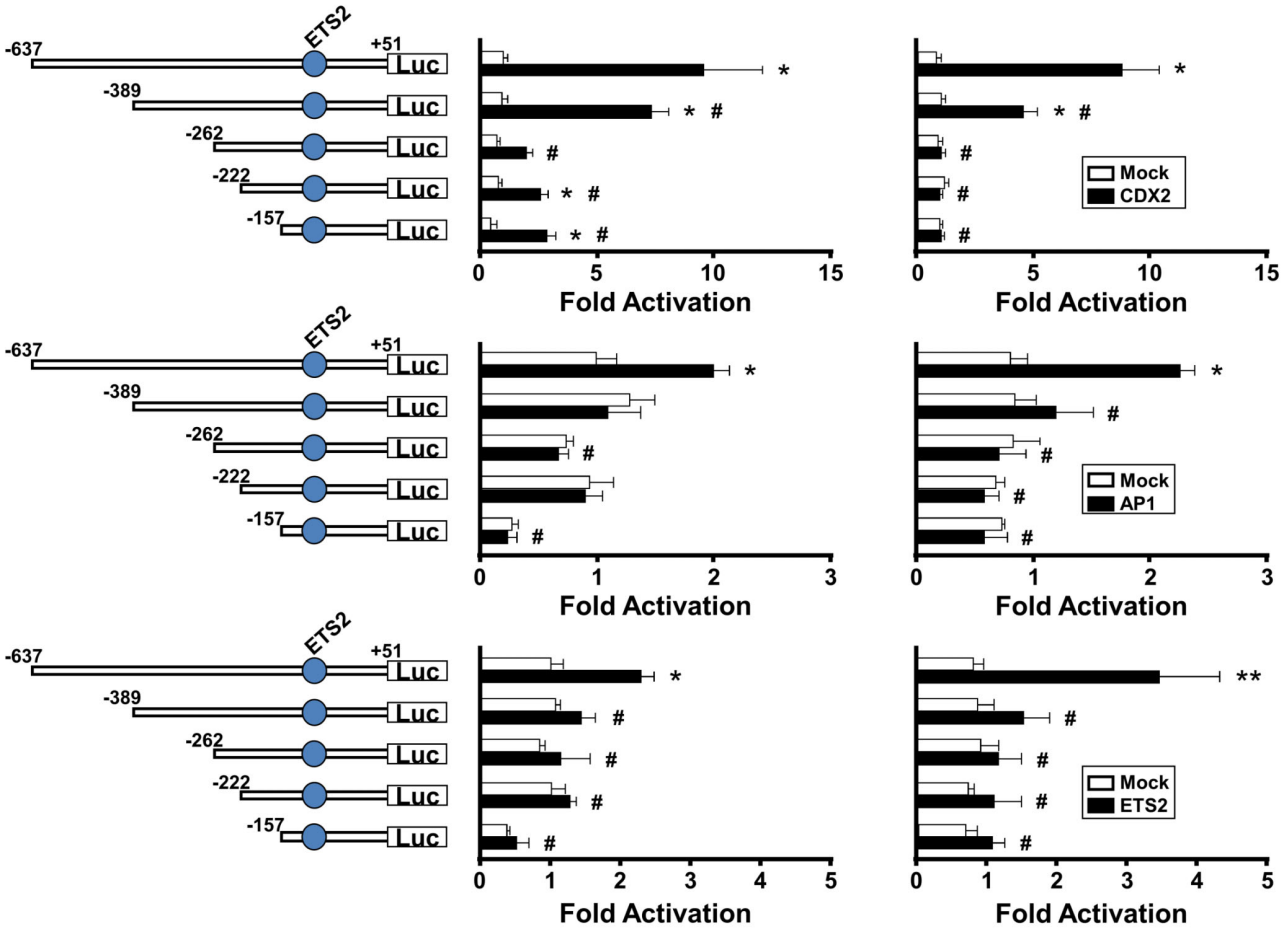
Examination of the effect of CDX2, AP-1 (JUN) or ETS2 on transcriptional activity of *IFNT1* and *IFNTc1* deletion constructs. A reporter construct containing various lengths of fragments from the upstream regions of *IFNT1* and *IFNTc1* genes were cotransfected into EF cells with *Cdx2* (upper) or AP-1 (*Jun*, middle), or *ETS2* (lower) expression plasmid, and the luciferase activity was determined. Transfection with the pSG5 (Mock) plasmid was used as an internal control. Results were expressed as the luciferase activity relative to that of the -637-*IFNT1* and -637-*IFNTc1*-reporter constructs (WT), respectively, without any expression plasmid (Mock). Values represent mean ± SEM from four independent experiments with replicate within each experiment. * and # indicate statistically significant difference in luciferase activity (*p* < 0.05) when compared to that of the control (wild type without an expression plasmid) and difference within the treated group, respectively.

To investigate whether deletions in the promoter regions of *IFNT1* and *IFNTc1* affected their transcriptional activity, the upstream regions between positions −637 and +51 were deleted at positions −389, −262, −222, and −157. To evaluate the roles of CDX2, JUN, or ETS2 on *IFNT1*- and *IFNTc1*-reporter transcription, each mutant reporter construct was cotransfected with *Cdx2*, *Jun*, or *ETS2* expression plasmid into EF cells. In the case of CDX2, the luciferase activities of -637-*IFNT1* and -637-*IFNTc1*-reporter constructs were increased 10-fold (Fig. 5 upper). Such high activities were decreased with −389 reporter constructs and further decreased with −262 reporter constructs of both *IFNT1* and *IFNTc1* genes. In the case of AP-1 (JUN), the luciferase activities of -637-*IFNT1*- and -637-*IFNTc1*-reporter constructs were increased approximately 2-fold (Fig. 5 middle). The reduction in luciferase activity was seen with −389 reporter constructs, and a second reduction in *IFNT1*-reporter construct was seen with −157. In the case of ETS2, the luciferase activities of -637-*IFNT1* and -637-*IFNTc1*-reporter constructs were increased 2 to 3-fold (Fig. 5 lower). The reduction in luciferase activity was seen with −389 reporter constructs, and further reduction was with −157 reporter of *IFNT1*. These results indicate that potential CDX2-binding sites exist at two locations between positions −637 and −389, and −389 and −262. Potential AP-1-binding sites are located between positions −637 and −389, and −222 and −157 of *IFNT1*-promoter. In addition to the previously reported ETS2 site at −79 [11], [35], a potential ETS2-binding site was located between −637 and −389 of both *IFNT1* and *IFNTc1* loci.

### Roles of CDX2-, AP-1-, or ETS2-binding on *IFNT* gene transcription

**Figure 6:**
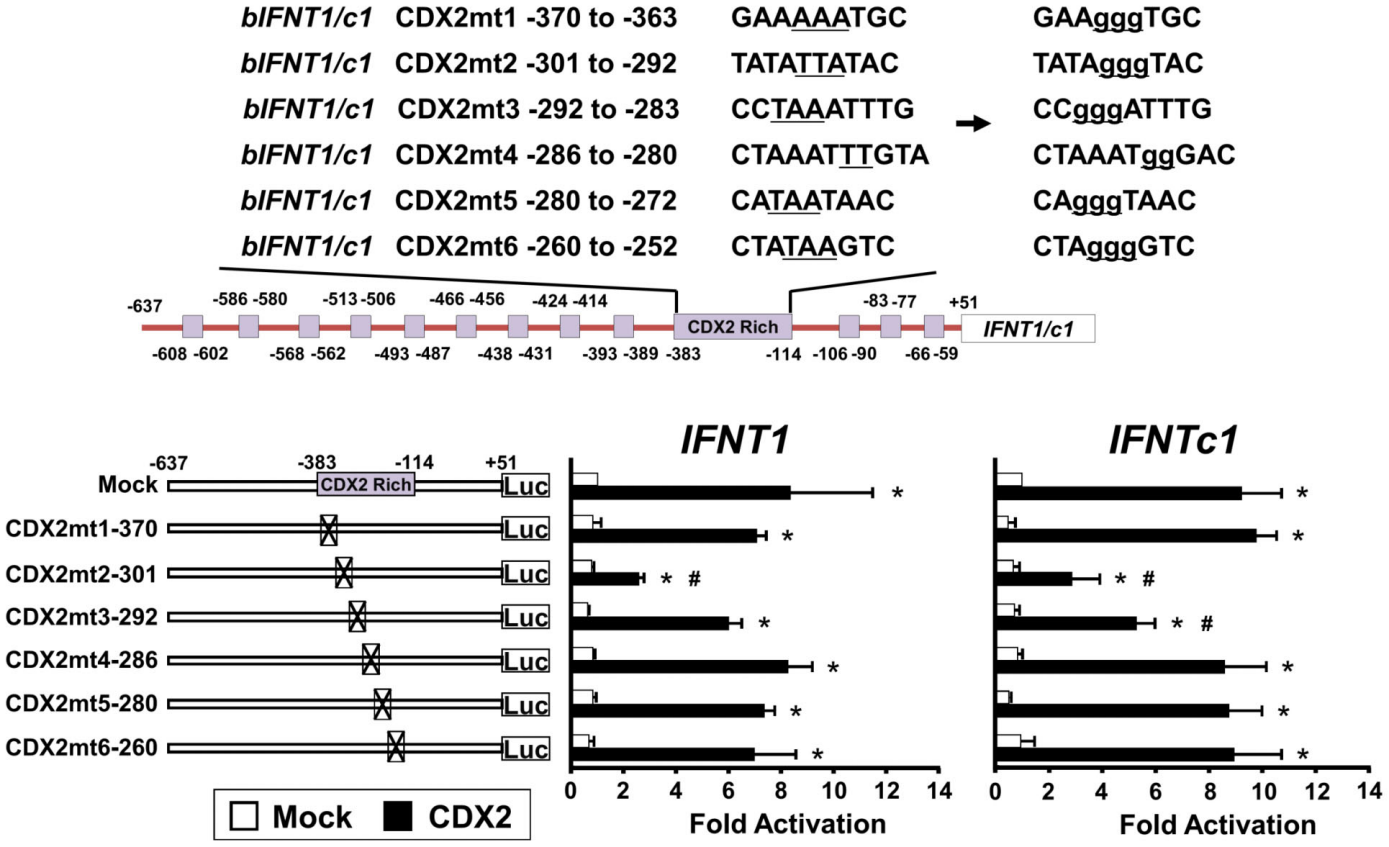
Transcriptional activity of the *IFNT1*- and *IFNTc1*-reporter constructs with point mutations at potential CDX-binding sites. Upper: Locations of CDX2 binding sites on the upstream regions of *IFNT1* and *IFNTc1* genes. Six mutation constructs (CDX2mt1 through CDX2mt6) were prepared: underlines indicate nucleotide changes in the wild type to mutated construct. Lower: The *IFNT1* and *IFNTc1* constructs, along with the pSG5 plasmid (Mock) or *Cdx2* expression plasmid, were cotransfected into EF cells, and the luciferase activity was determined. Results were expressed as luciferase activity relative to that of the -637-*IFNT1*- and -637-*IFNTc1*-reporter constructs (WT), respectively, without any expression plasmid (Mock). Values represent mean ± SEM from four independent experiments with replicate within each experiment. * and # indicate statistically significant difference in luciferase activity (*p* < 0.05) when compared to that of the control (wild type without an expression plasmid) and difference within the treated group, respectively.

**Figure 7:**
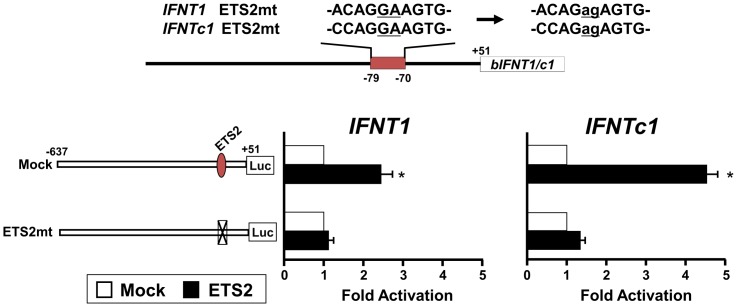
Transcriptional activity of the *IFNT1*- and *IFNTc1*-reporter constructs with point mutations at potential ETS-binding sites. Upper: Nucleotide changes in ETS2 site mutated construct (ETS2mt). Lower: The *IFNT1* and *IFNTc1* constructs, along with the pSG5 plasmid (Mock) or *ETS2* expression plasmid, were cotransfected into EF cells, and the luciferase activity was determined. Results were expressed as luciferase activity relative to that of the -637-*IFNT1*- and -637-*IFNTc1*-reporter constructs (WT), respectively, without any expression plasmid (Mock). Values represent mean ± SEM from four independent experiments with replicate within each experiment. *Statistically significant difference in luciferase activity (*p* < 0.05) when compared to that of the control (wild type without an expression plasmid).

**Figure 8:**
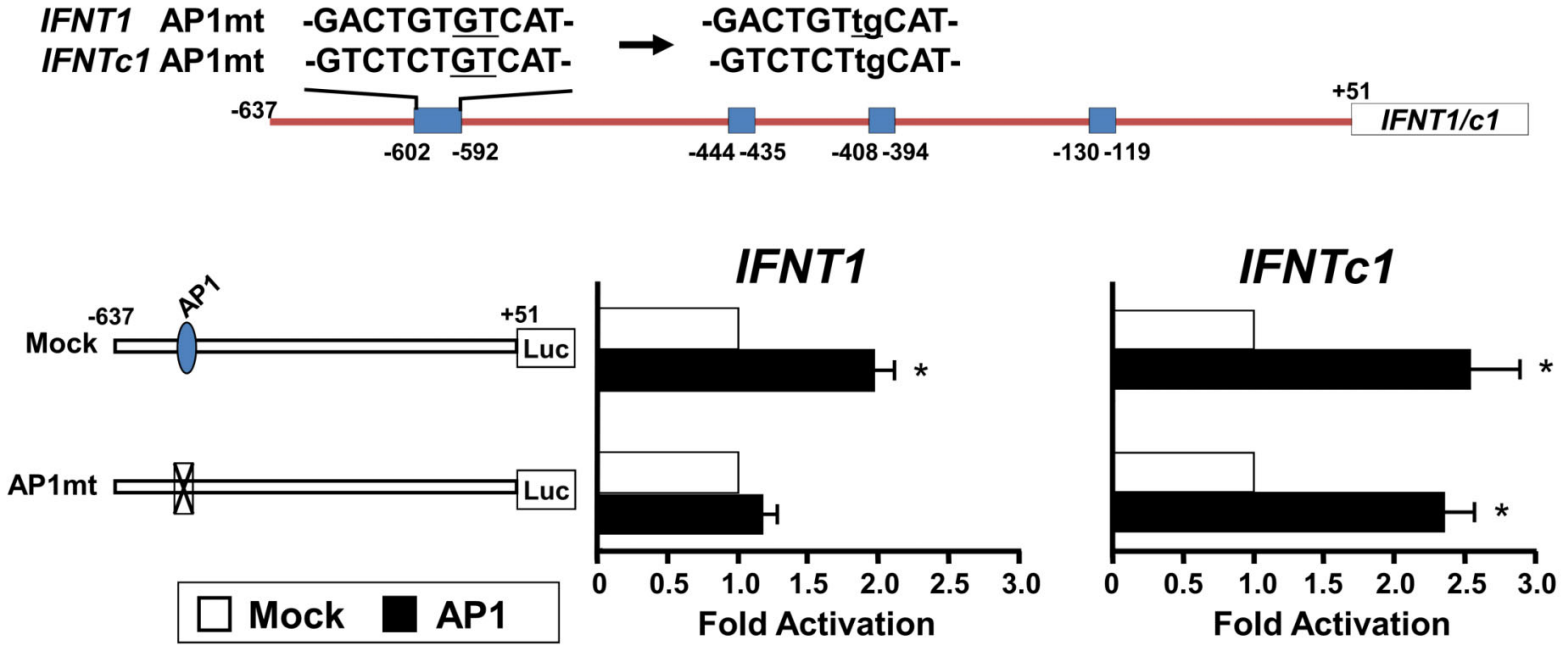
Transcriptional activity of the *IFNT1*- and *IFNTc1*-reporter constructs with point mutations at potential AP-1-binding sites. Upper: Nucleotide changes in AP-1 site mutated construct (AP1mt). Lower: The *IFNT1* and *IFNTc1* constructs, along with the pSG5 plasmid (Mock) or *Jun* expression plasmid, were cotransfected into EF cells, and the luciferase activity was determined. Results were expressed as luciferase activity relative to that of the -637-*IFNT1*- and -637-*IFNTc1*-reporter constructs (WT), respectively, without any expression plasmid (Mock). Values represent mean ± SEM from four independent experiments with replicate within each experiment. *Statistically significant difference in luciferase activity (*p* < 0.05) when compared to that of the control (wild type without an expression plasmid).

To investigate the requirement of CDX2, AP-1 (JUN), and ETS2 in transcriptional regulation of *IFNT1* and *IFNTc1*, we generated the reporter constructs bearing point mutations at potential binding sites for each of these transcription factors (Fig. 6, 7, 8, respectively). These mutant constructs along with *Cdx2*, *ETS2*, or *Jun* expression plasmids were then cotransfected into EF cells.

In both the *IFNT1*- or *IFNTc1*-reporter constructs, mutation of −301 to −292 (CDX2mt2) was effective in the down-regulation of luciferase activity (Fig. 6). The promoter activities with mutation at ETS2-binding site (*IFNT1*-ETS2mt and *IFNTc1*-ETS2mt) also decreased by 60% and 70%, respectively (Fig. 7), fully agreeing with the previous reports [11], [36]. Compared to mock, the activity of *IFNT1*-AP-1mt reporter decreased by approximately 50%; however, the transcriptional activity of *IFNTc1*-AP-1mt did not change (Fig. 8). These results indicate the existence of an AP-1-binding site between −602 and −592 of the *IFNT1* gene; however, such an AP-1-binding site in the *IFNTc1*-reporter construct was not located at the same position (Fig. 3).

## Discussion

Two forms of bovine conceptus *IFNT* transcripts *in utero* were found through the use of a next generation sequencer SOLiD3. It is well known that *IFN* type I genes are found in various mammals, but *IFNT* genes are found only in ruminants [3]. Based on the phylogenetic tree (Fig. 1 and Fig. S1), the *IFNT* clade shows a monophyletic relationship with *IFNW* genes. Considering the topology of the tree showing the cluster of *IFNT* and *IFNW* genes and the bootstrap value, *IFNT* can be originated from an *IFNW* gene by duplication in the ancestor species of ruminants. Indeed, these *IFN* type I genes are known to be located tandemly in the chromosome 8, suggesting that duplication events lead the emergence of several *IFNT* genes in the bovine genome [35]. Previously, a very careful examination was conducted to characterize polymorphic forms of bovine *IFNT* expressed by bovine conceptuses, identifying numerous *IFNT* genes, that are categorized into 3 groups [17], [18], [36]. These *IFNT* sequences were found from PCR amplified/cloned cDNA from a day 25 bovine conceptus cDNA phage library [37]. The fact that group 2 of bovine *IFNT* genes cannot be found in the bovine genome [18] suggests that new technologies other than PCR cloning need to be exercised to find a full spectrum of *IFNT* transcripts expressed by the bovine conceptuses. Expression levels of bovine *IFNT1* and *IFNTc1* found in this study represent those *in utero* during the peri-implantation period.

It has been well characterized that the proximal ETS2 site is very effective in the up-regulation of *IFNT1* transcription [11], [34]. In addition to the proximal ETS2-binding site, another ETS2 site may exist in the upstream region between −637 and −389. Regarding a trophectoderm specific CDX2 transcription factor, only one CDX2-binding site at −581 to −575 (distal CDX site) in the ovine *IFNT* gene has so far been identified and characterized [32]. Among numerous CDX2-like binding sites located on the upstream region of bovine *IFNT* genes, in addition to the distal CDX2, another CDX2-binding site could be located at −301 to −292 (Figs. 4 and 5). In this study, the far upstream AP-1 site mutation was effective in the down-regulation of *IFNT1*, but not *IFNTc1*, transcription. It was shown that a transcription factor JUN/CREBBP/ETS2 complex formation through the use of both distal AP-1- and proximal ETS2-binding sites is required for the maximum *IFNT* expression [8], [21], [31], [38]. Relatively lower levels of conceptus *IFNTc1* expression *in utero* may be linked to the absence of the distal −602 to −592 AP-1-binding site. These previous and present observations indicate that in addition to the upstream regions of *IFNT* genes initially characterized [11], [30−32], , more transcription factor binding sites could exist and function, particularly in the uterine environment. These results suggest that differences in the degree of conceptus *IFNT* transcription *in utero* could result from changes in the use of their transcription factor binding sites.

Recombinant IFNTs have been applied to cyclic or pregnant animals for extending inter-estrous intervals or improving pregnancy rates, respectively [5], [39], [40]. However, treatment effects with recombinant IFNT1 were found to be inconsistent. Based on the observations from this study, treatment with only one form of recombinant IFNT does not represent conceptus IFNT production in the uterus, which may explain the ineffectiveness of the IFNT treatment. Although the existence of other factor(s) cannot be excluded, a cocktail of two forms of IFNTs, IFNT1 and IFNTc1, with the appropriate concentration ratio, could be integral to successful treatment of inter-estrous interval extension or pregnancy improvement.

In conclusion, among numerous bovine *IFNT* genes, only two forms of conceptus *IFNT* genes expressed *in utero* were found in this study. Nucleotide sequences and their transcriptional regulation of the two *IFNT* genes are similar, but not the same, resulting in different degree of their expression *in utero*.

## Supporting Information

Figure S1 **Maximum likelihood phylogenetic tree of 218** ***IFNT*** **-related genes and 33 bovine** ***IFN*** **type I genes registered in Ensembl.** Amino acid sequences of 252 *IFNT* related genes including 33 bovine *IFNT* related genes in Ensembl were used for the phylogenetic analysis. Note that two Ensembl genes (ENSBTAT00000006179 and ENSBTAT00000023814) were not used in the study because of their short sequence length (112 and 68 aa, respectively). The procedure was the same as that of Figure 2. The percentage of 1,000 fast bootstrapping tests was shown if the value was ≥ 60%. Ensembl genes categorized as *IFNT*, *IFNW* and *IFNA* were colored in red, blue and green, respectively.
(PDF)

Table S1 **RPKM values for each of bovine** ***IFNT*** **-related genes.** Note: Among 35 bovine *IFNT*-related genes, eight genes were found as *IFNT* genes, from which two *IFNT* transcripts were found as those expressed *in utero* by the bovine conceptuses during the peri-implantation period.
(XLSX)

Table S2 A list of *IFNT*-related genes used in the phylogenetic analysis.
(XLSX)
