# Supplementary figures and images for: Transcriptional Regulation of Two Conceptus Interferon Tau Genes Expressed in Japanese Black Cattle during Peri-Implantation Period
Source: PLoS One. 2013 Nov 27;8(11):e80427. doi: 10.1371/journal.pone.0080427 (PMC3857836; doi:10.1371/journal.pone.0080427)

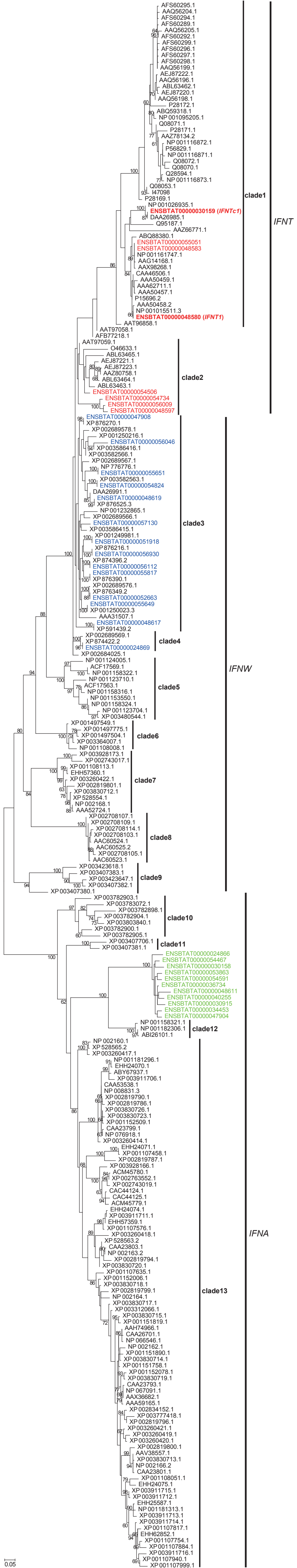

Supplement: Figure S1 — Maximum likelihood phylogenetic tree of 218 IFNT -related genes and 33 bovine IFN type I genes registered in Ensembl. Amino acid sequences of 252 IFNT related genes including 33 bovine IFNT related genes in Ensembl were used for the phylogenetic analysis. Note that two Ensembl genes (ENSBTAT00000006179 and ENSBTAT00000023814) were not used in the study because of their short sequence length (112 and 68 aa, respectively). The procedure was the same as that of Figure 2. The percentage of 1,000 fast bootstrapping tests was shown if the value was ≥ 60%. Ensembl genes categorized as IFNT, IFNW and IFNA were colored in red, blue and green, respectively. (PDF) [file pone.0080427.s001.pdf]
